# Treatment with Synthetic Glucocorticoids and the Hypothalamus-Pituitary-Adrenal Axis

**DOI:** 10.3390/ijms18102201

**Published:** 2017-10-20

**Authors:** Rosa Maria Paragliola, Giampaolo Papi, Alfredo Pontecorvi, Salvatore Maria Corsello

**Affiliations:** Unit of Endocrinology, Università Cattolica del Sacro Cuore, Largo “A. Gemelli” 8, I-00168 Rome, Italy; rosamariaparagliola@gmail.com (R.M.P.); papigiampaolo@hotmail.com (G.P.); alfredo.pontecorvi@unicatt.it (A.P.)

**Keywords:** synthetic glucocorticoid, tertiary hypoadrenalism, iatrogenic Cushing’s syndrome

## Abstract

Chronic glucocorticoid (GC) treatment represents a widely-prescribed therapy for several diseases in consideration of both anti-inflammatory and immunosuppressive activity but, if used at high doses for prolonged periods, it can determine the systemic effects characteristic of Cushing’s syndrome. In addition to signs and symptoms of hypercortisolism, patients on chronic GC therapy are at risk to develop tertiary adrenal insufficiency after the reduction or the withdrawal of corticosteroids or during acute stress. This effect is mediated by the negative feedback loop on the hypothalamus-pituitary-adrenal (HPA) axis, which mainly involves corticotropin-release hormone (CRH), which represents the most important driver of adrenocorticotropic hormone (ACTH) release. In fact, after withdrawal of chronic GC treatment, reactivation of CRH secretion is a necessary prerequisite for the recovery of the HPA axis. In addition to the well-known factors which regulate the degree of inhibition of the HPA during synthetic GC therapy (type of compound, method of administration, cumulative dose, duration of the treatment, concomitant drugs which can increase the bioavailability of GCs), there is a considerable variation in individual physiology, probably related to different genetic profiles which regulate GC receptor activity. This may represent an interesting basis for possible future research fields.

## 1. Introduction

Named for their effect on carbohydrate metabolism, glucocorticoids (GCs) regulate different cellular functions as homeostasis, metabolism, development, cognition, and inflammation [1]. Furthermore, GCs have a critical role in adaptation to environmental changes, stress response, and immune modulation [2,3,4].

In consideration of their anti-inflammatory action and immunosuppressive activity, chronic GC therapy is widely prescribed in the world and represents the basis for the treatment of numerous inflammatory and autoimmune diseases [5,6,7].

Synthetic GCs are available in different formulations (oral, intravenous or intramuscular, inhalation and topical). Unfortunately, their therapeutic actions are counterbalanced by adverse side effects associated with high doses and long-term use, such as osteoporosis, skin atrophy, diabetes, hypertension, abdominal obesity, acne, infection, and growth retardation [8], as well as ocular hypertension [9]. Indeed, the so called “iatrogenic Cushing’s syndrome” related to exogenous administration of GCs, represents the most frequent cause of hypercortisolism [10,11]. Moreover, patients in treatment with synthetic GCs are at risk to develop central adrenal insufficiency related to the inhibition of the hypothalamus-pituitary-adrenal (HPA) axis. Supraphysiologic GC doses inhibit both corticotropin-release hormone (CRH) production and pituitary adrenocorticotropic hormone (ACTH) release. When this inhibition persists longer than the duration of GC exposure, it is defined as adrenal suppression [12].

Due to their large therapeutic diffusion, synthetic GCs treatment represents one of the most frequent causes of secondary adrenal insufficiency [13]. This is a life-threatening condition, in particular after the withdrawal of GCs, and requires steroid replacement therapy to avoid the risk of acute adrenal insufficiency. Suppression of endogenous ACTH can result in adrenocortical hypoplasia or atrophy and, after GCs withdrawal, ACTH depression may take longer to pass off [14]. Even after ACTH secretion has been fully restored, the hypoplastic adrenal glands may require a long time to return to normality. Adrenal gland recovery time can be short (few days) [15], or as long as several weeks [16] or even more. The results from a recent systematic review show that hypoadrenalism persists in 15% of patients retested three years after GC withdrawal [17].

The aim of this review is to evaluate the physiopathology and the clinical effects of synthetic GCs on HPA function.

## 2. Normal Physiology of HPA Axis

GCs are steroid hormones synthesized and released by the adrenal glands in a circadian manner, in response to physiological cues and stress, and controlled by the main circadian oscillator located in the suprachiasmatic nucleus (SCN) of the hypothalamus [18]. In healthy subjects with normal nocturnal sleep and daytime wakefulness, late evening cortisol between 11:00 p.m. and 1:00 a.m. is very low or undetectable. Then cortisol levels increase overnight to reach a peak in the early morning (between 6:00 a.m. and 9:00 a.m.), and gradually decline slowly throughout the day [19]. The increase in cortisol secretion starting in the early morning helps to maintain plasma glucose (via increases in hepatic gluconeogenesis) until awakening when the overnight fast is broken [20]. No difference in physiological cortisol secretion is related to weight or gender [21]. Moreover, the correlation with age (which is positive for melatonin acrophase secretion), is negative for cortisol secretion. This suggests both weakened responsiveness of the circadian system in the elderly to the day-night cycle and also a different relationship between the pacemakers driving melatonin and cortisol circadian secretion [22].

**Figure 1:**
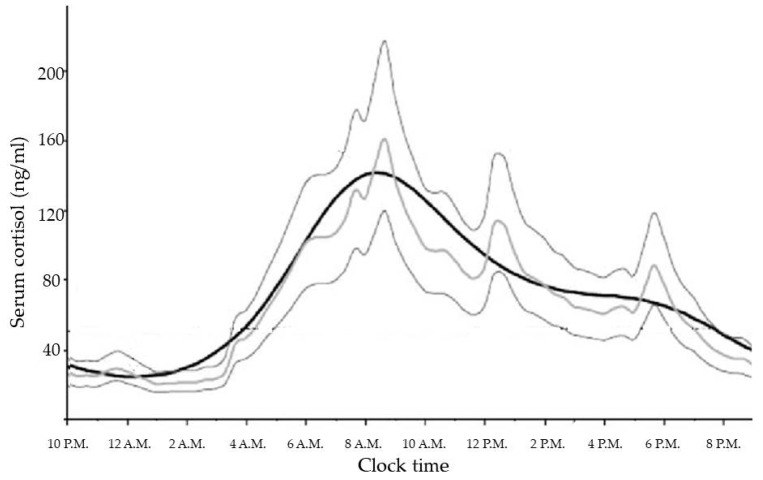
Daily cortisol rhythm in healthy volunteers [23].

The cortisol production rate is based on a number of secretory episodes occurring during the 24 h period. The minimal secretory activity, during which cortisol secretion is minor, occurs between four hours prior to and two hours after sleep onset; a preliminary nocturnal secretory episode occurs three to five hours after sleep, while a main secretory phase occurs during the six to eight hours of sleep and continues through the first hour of wakefulness. Then, an intermittent waking secretory activity of four to nine secretory episodes can be found in the 2–12 h waking period [19] (Figure 1).

The measurement of the total daily amount of cortisol production has been demonstrated to be around 5.7–7.4 mg/m^2^/day or 9.5–9.9 mg/day, supporting the utility of a therapeutic regime with oral daily hydrocortisone doses (15–25 mg/day) lower than thought in the past [24].

GC release is regulated by the activity of the HPA axis. Basal and stress-inputs to the hypothalamic parvocellular nuclei lead to an increase in CRH release into the hypophyseal-portal veins. The most important basal input to CRH secretion derives, in turn, from the circadian rhythm generator in the hypothalamic SCN. Arginine vasopressin (AVP) that is co-expressed in some CRH neurons also acts as an ACTH secretagogue. AVP alone has only a slight releasing activity by itself, but it is a potent synergistic releaser in combination with CRH and its role is physiologically relevant in the stress response [25].

The SCN is composed of around 10,000 neurons located above the optic chiasma and it is divided into two regions: a ventral “core” region receiving information from the retina and brain stem and a dorsal “shell” region, which represents a primary pacemaker driving behavioural and other rhythms [26]. The phase of the circadian rhythm is synchronized on the basis of the phase of the day-night cycle to which it is exposed. Photic environmental cues are perceived by melanopsin-containing retinal ganglion cells that transfer their outputs to the SCN via the retinohypothalamic tract, whose main neurotransmitter is glutamate [27]. CRH release from the paraventricular nucleus (PVN), regulated by the input from the central pacemaker, stimulates the release of ACTH from the corticotroph cells in the anterior pituitary and then ACTH in turn promotes cortisol secretion from the adrenal cortex. As well as SCN, important excitatory inputs for CRH secretion also originate from the amygdala and the raphe nuclei (sites of origin of the serotonergic projections) and from the locus coeruleus of the brain stems, where ascending noradrenergic fibers originate [25]. The most important neurotransmitters acting on CRH release are serotonin, acetylcholine, catecholamines, and neuropeptide Y, but CRH neurons are also stimulated by the immune system via prostaglandins and several cytokines. Inhibitory inputs on CRH secretion derive from the hippocampus and the locus coeruleus of the midbrain and are mainly mediated by gamma-aminobutyric acid (GABA) and nitric oxide (NO) [28].

CRH regulates ACTH secretion in two ways; it acutely stimulates the corticotrophs to release the already-stored ACTH into the pituitary venous effluent and then, through the inferior petrosal sinuses, into the internal jugular veins. Moreover, CRH also promotes the synthesis of new ACTH by activating gene transcription for its precursor molecule proopiomelanocortin (POMC) and its post-translational processing [29].

ACTH acts on adrenal glands to stimulate cortisol release via melanocyte type-2 receptor (MC2R) expressed on the zona fasciculata and zona reticularis [30]. This mechanism is mediated by a G-protein activity, which increases intracellular cyclic adenosine monophosphate (cAMP) (second messenger) promoting the liberation of steroidogenic acute regulatory (StAR) protein. StAR protein, in turn, mediates the translocation of cholesterol to the inner mitochondrial membrane, where the first steroidogenic enzyme is located [18]. The activity of the cytoplasmic StAR protein represents a rate-limiting process for adrenal steroidogenesis.

The adrenal gland also contains circadian clock genes, expressed in the zona glomerulosa and zona fasciculata, and regulated by the splanchnic nerve, that establish specific time intervals during which the adrenal cells are most responsive to ACTH stimulation [31]. However, the expression of adrenal clock genes show a six-hour phase delay in relation to the SCN [19].

After the release in the blood, cortisol circulates in “free” form (~5–6% of the total plasma cortisol, which diffuses into the cells exerting its biological effects on target tissues) or bound primarily to two proteins. Cortisol can significantly bind to the “low affinity-high capacity protein” albumin when its secretion rate is high; in physiological secretion rates, cortisol has a specific carrier, the “high affinity-low capacity” corticosteroid binding globulin (CBG or transcortin) produced by the liver [29]. Currently available assays measure total cortisol concentration and not the biologically-active free cortisol; this can lead to inaccurate interpretation of the results in presence of CBG alterations (for example, high concentrations of CBG in women receiving oral estrogens or in pregnancy could cause an increase of total cortisol concentration while, falsely, hypocortisolism can be detected in presence of low CBG in cirrhotic patients) [24].

A negative feedback loop exerted by cortisol secretion has inhibitory effects at pituitary and hypothalamic levels, but there is no feedback on the SCN [32].

## 3. Effects of Glucocorticoids Therapy on the Hypothalamus–Pituitary–Adrenal Axis

### 3.1. Synthetic Glucocorticoids

Considering the wide use of synthetic GCs, many patients could potentially be at risk of developing adrenal insufficiency. Recent studies evaluate that, in 2008, almost 1% of the UK adult population were exposed to oral GCs (including 0.79% on long-term courses, longer than three months) [33,34].

In Table 1 the most used synthetic GCs and their characteristics have been reported.

Even if data about the clinical impact of these medications are limited, about 6% of hospitalized patients may have a GC-induced adrenal insufficiency [35]. Interestingly, novel treatments aimed at improving the benefit/risk ratio of GCs therapy (selective GC receptor agonists, SEGRAs), which are currently being trialed, are unlikely to alter the risk of adrenal insufficiency. This is probably because adrenal insufficiency can develop with the same mechanism, mediating the “therapeutic” anti-inflammatory effects of these compounds [36]. Furthermore, it also has to be considered that the “local” use of GCs (inhalation, intra-articular, or cutaneous) is increasingly widespread in clinical practice and that their potential systemic effects should not be underestimated.

#### 3.1.1. Inhaled Glucocorticoids

In the early 1970s, beclomethasone dipropionate was proposed as the first inhaled GC: this therapy represents the mainstay of maintenance treatment of persistent asthma and is recommended as the initial therapeutic option in children and adults. Since then, several new compounds have been synthesized (ciclesonide, budesonide, fluticasone, momethasone) (Table 2).

The introduction of these compounds has considerably reduced the dose of GCs required for the treatment of inflammation and it was hypothesized to reduce the risk of systemic adverse effects due to the localized site of administration. However, this is not fully true: in fact, after inhalation, the amount that reaches the lungs is absorbed into the bloodstream and, in most cases, it is not metabolized to biologically-inactive compounds by the respiratory tract [37]. The remaining portion of the inhaled compound is usually swallowed, absorbed by the gastrointestinal tract, and it can remain bioavailable after the first liver pass. These compounds at low-to-moderate doses are generally considered safe; however, high doses used for a prolonged time may be associated with a risk of systemic effects. A systematic review and meta-analysis evaluates the effects of inhaled GCs on endogenous cortisol suppression in terms of urinary cortisol suppression. The strongest dose-response for urinary cortisol suppression is observed in patients treated with beclomethasone, followed by fluticasone and budesonide, while no significant urinary cortisol suppression was associated with ciclesonide treatment. However, although ciclesonide does not affect cortisol levels, this appears to be due to its unique pharmacokinetic properties rather than the use of a novel formulation [38].

#### 3.1.2. Intra-Articular Glucocorticoids

Intra-articular GC injections are routinely used for the localized treatment of joint pain, in particular for knee and shoulder [40]. This therapy is typically indicated for the treatment of rheumatoid arthritis, osteoarthritis, crystalline arthropathies, or other inflammatory arthropathies after the failure of conventional therapy in the control of pain. Even if the administration of GCs is normally confined to the intra-articular cavity, systemic absorption has been widely demonstrated, as confirmed by the beneficial effects on other joints that have not been injected [41]. The most used compounds are triamcinolone acetonide, triamcinolone hexacetonide, and methylprednisolone acetate, but betamethasone acetate, betamethasone sodium phosphate, as well as dexamethasone, have been also used [42].

The true incidence of tertiary hypoadrenalism with the use of these local compounds is largely unknown and based mainly on case reports while guidelines concerning the frequency and interval between injections are lacking [43]. However, a recommendation of up to three GC intra-articular injections per year with a minimum of thirty days between injections has been advocated to avoid HPA axis suppression [42]. A single dose may be enough to cause a “biochemical” although not “clinical” HPA axis suppression, while patients receiving repeated intra-articular steroid injections can develop adrenal insufficiency after withdrawal of the treatment. Recovery of the HPA axis to baseline normally requires 1–4 weeks, but can be longer in consideration of the dose and frequency of injections [41].

It is important to underline that a non-recognized adrenal suppression can be extremely dangerous for the juvenile population who may not be aware of the signs and symptoms, as well as for athletes, and for those who participate in extreme sports at risk of trauma, infection, and acute stress [44].

#### 3.1.3. Topical Corticosteroids

Topical GCs (Table 3) are normally absorbed through normal skin, especially in inflammatory and occlusive skin. Clobetasol propionate is one of the most potent topical GCs: its potency is estimated to be 600 times higher than hydrocortisone [45] and the use of 2 g/day of 0.05% clobetasol propionate can decrease morning cortisol levels after a few days, while with 100 g/week iatrogenic Cushing’s syndrome and hypoadrenalism can appear [46,47].

Iatrogenic Cushing’s syndrome with consequent HPA suppression due to topical corticosteroid use has been reported by several authors [48]. Side effects can be caused by the direct absorption through ulcerate skin, but also by the oral mucous membrane or by unintentional ingestion, and they are more frequent in pediatric age. Old data collected in 1986 showed a positive relationship between adrenal suppression and the increase in serum cortisol level following the use of topical 1% hydrocortisone; furthermore, the HPA axis suppression was longer in infant compared with older children, because infants have a larger ratio body surface area to body weight [46]. This is the reason why potent topical GCs should be avoided in children under the age of 12 years [45].

### 3.2. Negative Feedback Loop and Glucocorticoid Receptors

In physiological conditions, the degree of feedback inhibition by GC on the central nervous system and on pituitary sites, in combination with the hypothalamic neuropeptide release, defines the set point for plasma GC levels. Prolonged supraphysiologic GC administration impairs the ability of the hypothalamus and pituitary to respond to acute GC withdrawal, resulting in iatrogenic adrenal insufficiency.

The most important inhibitory effect of synthetic GCs on the HPA axis is mediated by the negative feedback loop which, in physiological conditions, is exerted by cortisol and is mediated by GC receptors. Indeed, cortisol, like other steroid hormones, interact with cytoplasmatic receptors which cause changes in gene transcription and translation [49].

In this context, the two main receptors of interest are the “lower affinity-higher capacity” GC receptor (GR) and the “higher affinity-low capacity” mineralocorticoid (MC) receptor (MR), also called type II and type I receptors, respectively [50].

Type I MR has high affinity for aldosterone, cortisol, and corticosterone, and three- to five-fold lower affinity for the synthetic GC dexamethasone. Furthermore, the proportion of inactivated type I MRs increased (55–65%) after dexamethasone treatment. Type II GR has high affinity for dexamethasone and three- tofive-fold and 10- to 20-fold lower affinity for corticosterone and for aldosterone respectively [51].

MR expression shows a restricted tissue distribution, with high expression in classical aldosterone target organs, such as kidney, colon, salivary glands, and specific brain regions, and more modest expression in heart, vascular tissues, adipocytes, and specific immune cell populations [52].

In contrast, GR are more widely expressed in many peripheral tissues than MR. In some tissues, such as distal tubules of the kidney, the action of 11-β-hydroxysteroid dehydrogenase type 2 (11β-HSD2), which converts the active cortisol into inactive cortisone, “protects”s MRs from the cortisol. This is the reason why the primary activity in regulation of sodium-potassium balance and blood pressure in distal tubules is exerted by aldosterone, even if the concentration of serum cortisol is higher. However, this mechanism can be unbalanced in conditions of overexposure to GCs that override 11β-HSD2 activity, as observed in hypertension related to Cushing’s syndrome [29].

In vitro assay demonstrates that GRs and MRs respond to different levels of GC, suggesting that, together, they confer a larger dynamic range of sensitivity to these hormones [53].

Due to higher levels of MR expression, supported by the anatomical distribution of MR-mRNA determined by in situ hybridization histochemistry, the brain is more sensitive than the corticotrophs to GCs [53]. Low basal corticosterone levels activate both MRs and GRs in the hippocampus, whereas pituitary is very insensitive, as evidenced by a failure of acute stress levels of endogenous GCs to activate GRs in the pituitary [51]. Then, GRs in the brain, and especially in the hippocampus, are more sensitive to circulating levels of GCs than the pituitary [51].

Although some MRs are present in the anterior pituitary, studies indicate that GR-mediated effects are predominant in the pituitary [54], while inhibition of the brain–pituitary unit depends mainly from MRs [55,56].

Models of GR disruption in specific brain regions help to uncover the multiple roles of the GR in regulation of the HPA axis. Mice with a specific GR-deletion in the forebrain show HPA axis hyperactivity; basal cortisol is increased, as well as post-stress cortisol and ACTH levels [57]. Mice carrying GR-deletion in the PVN show a significant decrease in GR-protein in the PVN, but normal levels in the pituitary and adrenal glands. Elevated CRH immunoreactivity in the PVN, and elevated concentrations of plasma ACTH and cortisol are present. Pituitary GR-deletion is associated with HPA axis hyperactivity and increasing ACTH and cortisol levels. On the other hand, MR-deletion in the forebrain causes upregulation of GR expression in the hippocampus, but no effect in basic synaptic transmission or in circadian or post-stress cortisol concentrations [57].

Brain input is required for appropriate ACTH responses to GC feedback. Comparison of brain and pituitary sensitivity to feedback in animal models has been investigated in male rats. In animal models with lesions of medial basal hypothalamic or para-ventricular nuclei, ACTH fails to respond to GC feedback. In these models without hypophysiotrophic input, supraphysiologic GC levels are required to inhibit exogenous CRH-induced ACTH secretion [58].

**Figure 2:**
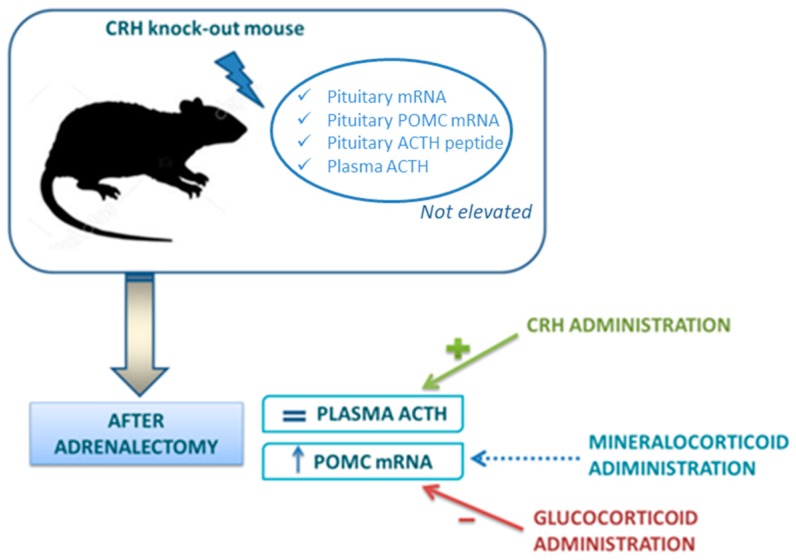
Corticotropin-release hormone (CRH) knock-out (KO) mouse model in regulation of hypothalamus-pituitary-adrenal (HPA) axis [59]. In CRH KO models proopiomelanocortin (POMC) mRNA, but not plasmatic adrenocorticotropic hormone (ACTH), rises after adrenalectomy. Glucocorticoids, but not mineralocorticoids (blue dotted arrow) reduce POMC increase induced by adrenalectomy. CRH administration restores a significant ACTH secretion.

The experimental model of CRH knockout (KO) mice has provided a unique system to define the role of CRH in regulation of the HPA axis [59]. In these animals, basal pituitary POMC mRNA, ACTH peptide content within the pituitary and plasma ACTH concentrations are not elevated. If adrenalectomy is performed, POMC mRNA content rises and this increase is reversed by GC replacement, but not by aldosterone. However, in contrast to POMC mRNA, plasma ACTH does not increase after adrenalectomy: only the administration of CRH to adrenalectomized CRH KO mice can restore a significant ACTH secretion. These interesting findings demonstrate that the increase of POMC gene expression depends by the loss of GC feedback, but the secretion of ACTH depends essentially on CRH (Figure 2).

In fact, ample pituitary stores of ACTH in the CRH KO mice have been demonstrated but CRH administration is essential for its release. Moved to clinical practice, this model helps to understand that, in adrenal insufficiency, loss of GC feedback, by itself, can increase POMC gene expression in the pituitary (suggesting that CRH is not absolutely required for changes in POMC expression in response to absolute GC deficiency), but CRH action is essential to guarantee the increase of ACTH secretion. This can also explain why, after withdrawal of chronic GC treatment, reactivation of CRH secretion is a necessary prerequisite for the recovery of the HPA axis. CRH is required for posttranscriptional events necessary for the release of ACTH and for trophic support of the adrenal glands [59]. Indeed, CRH deficiency results in adrenal atrophy and hyporesponsiveness to a variety of stressors.

The normal ACTH responses to CRH stimulation test in patients being withdrawn from GC treatment further confirms that the suppression is likely due to an ongoing deficit in CRH—rather than in ACTH secretion [60].

Furthermore, GR-mediated feedback inhibition on the pituitary only occurs at chronic GC levels that incur adverse peripheral effects [5]. Consequently the inhibition of endogenous cortisol secretion is an important marker of systemic activity by synthetic GCs. In physiological conditions, cortisol can both directly inhibit POMC transcription and ACTH secretion and also indirectly inhibit ACTH secretion by decreasing CRH secretion [50] which, as mentioned before, represents an important regulator of ACTH release.

Synthetic GCs, in turn, inhibit pituitary POMC expression through the binding of GR to a negative promoter on the POMC gene [61]. In the absence of its ligand, GR is retained in the cytosol in a chaperone-containing multiprotein complex, which maintains affinity for the ligand. Upon hormone binding, GR translocates to the nucleus acting as a transcription factor. The GR subunits homodimerize and bind DNA at GC response elements (GREs) close to target genes. GRE-bound GR recruits multiple transcriptional co-activator complexes, which stimulate transcription [61].

Moreover, also a variety of transcription factors can be involved in these mechanisms [29]. GC feedback inhibition occurs in several time domains, referred to as fast, delayed (or intermediate), and slow feedback [62]. The so-called “fast feedback” is a rapid-non genomic mechanism of action, which has been well documented in rats [63], although there is evidence suggesting a similar mechanism in humans [64].

Previous studies on negative feedback in humans have predominately used the potent GC agonist dexamethasone to inhibit the HPA axis activity over long time: in the clinical practice, this mechanism is used to confirm the diagnosis of Cushing’s syndrome, characterized by the loss of the capacity of response of HPA axis to GC feedback [65]. However, the literature on the mechanism of rapid GC inhibition of HPA activity is confusing: in rats, there is in vivo evidence that this involves a MR-mediated process [63], but ex vivo studies suggest no MR involvement [66]. The main limitation of the studies is that they do not consider the feedback from endogenous pulses of cortisol, which act in a faster time domain and activate both GRs and MRs.

Other evidence suggests that in man both GRs and MRs are involved in negative feedback, with GRs effecting a rapid non-genomic feedback in anterior pituitary and MRs sensing higher GC levels while levels are still rising. In this perspective, both receptors seem to be involved, exerting different temporal effects and providing a feedback sensor mechanism with a wide range of sensitivity. This fast feedback inhibition is mainly GR dependent, is independent from CRH drive, occurs predominately in the anterior pituitary and only blocks the regulated pathway of ACTH release [67]. In fact, circulating POMC peptide concentrations are unaffected, suggesting that the rapid feedback effect is definitely targeted on ACTH secretion and is only reduced by GRs antagonist pretreatment, and not by MRs antagonist.

The delayed feedback and the slow feedback, reflecting chronic exposure to GCs over days or weeks, affect both basal and stimulated hypothalamic–pituitary activity. In fact, when chronic GC exposure exceeds the 24-h mean cortisol production, the capacity of the HPA axis first to respond to stimulation, and ultimately to maintain basal activity, is suppressed [50]. ACTH levels and its antiapoptotic effects on the adrenal cortex are inhibited.

Delayed feedback is most effective at 1–2 h while slow feedback becomes manifested over several hours. This mechanism mainly occurs at pituitary level and, also in this case, is exerted via type II GRs [68].

Posterior pituitary AVP is also responsive to GC levels in dogs and humans [69]. Basal concentration of AVP mRNA in the CRH KO mice are elevated, accordingly with other studies in rats which demonstrated an elevation in parvocellular CRH mRNA with reduction of plasma corticosterone concentrations [70].

Adrenalectomy in CRH KO mice determines a further increase in hypothalamic AVP mRNA, demonstrating their ability to respond to the absolute GC deficiency in a manner similar to that of wild-type mice [59]. In CRH KO mice different expression in AVP and POMC gene between adrenalectomized and intact animals has been observed [59]. This is probably due to the suppressive effects of aldosterone, to the increased sensitivity to low level GC concentrations or to the normal regulation of these endpoints by low GC levels [71].

Since it is very difficult to establish in vivo anti-inflammatory and MC action of synthetic GCs, in their studies Grossmann et al. [65] evaluated these characteristics by employing a human GR (hGR)-dependent transactivation assay in vitro (CV-1 cells). Then, they compare MC potencies of different synthetic corticosteroids in an equivalent assay differing for the presence of the human MR (hMR) instead of the hGR. Even if some results are congruent with GC and MC potency lists reported in the literature, some important considerations are to be due. For example, despite the fact that the GC potency of dexamethasone in vivo is reported to be slightly higher, or equal, to that of betamethasone, the authors observe a moderately higher transactivation activity of betamethasone compared with dexamethasone. Furthermore, it has been confirmed that aldosterone (which is often described as possessing no GC activity) shows GR-mediated transactivation at concentrations relative to cortisol that are not even reached neither in primary hyperaldosteronism nor in replacement therapy with fludrocortisone [65].

The biological activity of cortisone and prednisone depends on first pass activation by hepatic 11β-hydroxysteroid-dehydrogenase type 1 (11β-HSD1) [72]. This is the reason why, when these compounds are injected in a tissue without 11β-HSD1, they have no biological activity.

Regarding MC potency, there is less data. First of all, it is well known that, besides transactivation, MC activity in target tissues is regulated by 11β-HSD2, which is co-localized with the MRs and inactivates 11-hydroxysteroids to their corresponding 11-oxoderivatives [73]. Furthermore, the presence of the 6α-methyl group diminishes the MC activity in vivo, as shown for 6α-methylprednisolone, which presents lower MC activity, but higher GC activity compared with prednisolone [65].

Deflazacort exhibits a very low MC activity, supporting the opinion that it causes less MC-side effects than some of the older steroids. However, other studies confirm that this compound is rapidly metabolized to desacetyldeflazacort [74] which, in turn, shows some MC activity [65].

However, the mechanisms regulating the activity of synthetic GCs are more complex: it is mandatory to consider, in fact, that the receptor binding is only a prerequisite of the more delicate process of transactivation, and that there is not always a correlation for all compounds between “binding affinities” and “transactivation” [65]. For example, regarding triamcinolone, its relatively low receptor affinity, both for MRs and GRs, contrasts with its high in vivo potency [65].

Furthermore, in vitro systems differ from in vivo conditions in several aspects (steroid metabolizing enzymes and different concentrations of heat shock proteins).

### 3.3. Clinical Consequence: Tertiary Hypoadrenalism and Adrenal Crisis

**Figure 3:**
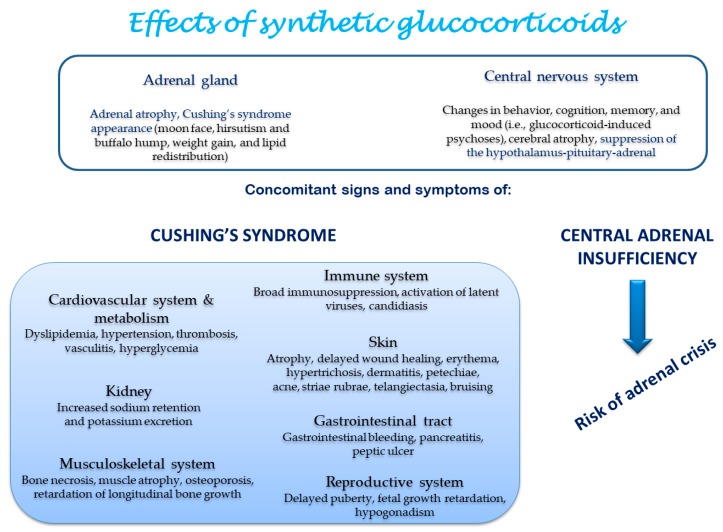
Tissue side effects of synthetic glucocorticoids [1].

The systemic effects induced by synthetic GCs are reported in Figure 3.

Several studies have evaluated the occurrence of hypoadrenalism and adrenal crisis induced by GCs, focusing on patients treated with inhaled and topical GCs and on those treated with high-dose GCs for hematological and rheumatic diseases. Furthermore, studies concerning HIV-infected patients are focused on the possible interaction between GCs and antiretroviral drugs [75,76,77]. However, in consideration of substantial differences in diagnostic criteria for adrenal insufficiency, the assessment of a clear incidence is not possible. The most important bias is represented by the variability in methods used for adrenal insufficiency evaluation: ACTH test is the most used, both with standard and low doses, but other tests, including insulin tolerance tests and CRH stimulation, have been used [17]. The choice of the most appropriate test for the assessment of HPA axis alterations remains largely debated and, for each test, different cut-offs have been reported with different degrees of sensitivity and specificity [24]. Prevalence estimates from some of the largest observational studies range from 14–63% [17].

Side effects on HPA function are more common in patients who are taking other medications that can alter the pharmacodynamics of GCs and potentiate their effects.

The concomitant use of drugs which act on cytochrome P450 3A4 activity can increase the bioavailability of synthetic corticosteroids worsening both iatrogenic Cushing syndrome and the degree of inhibition of the HPA. Several case reports involving HIV-infected patients treated with the concomitant use HIV protease inhibitor ritonavir (RTV) and triamcinolone have been reported [75]. Interestingly, the discontinuation of ritonavir promotes the clearance of the elevated triamcinolone serum levels, restoring HPA axis activity. Other cases of interaction leading to iatrogenic Cushing’s syndrome and tertiary hypoadrenalism have been described in HIV patients between ritonavir and inhaled GCs [76], but also, with the same mechanisms, in patients treated with intra-articular [42] or topical GCs who were taking protease inhibitors, itraconazole, macrolides, and diltiazem [45].

Furthermore, different factors can influence the pharmacokinetics of GCs. A study examining 54 patients of varying ages in treatment with oral and intravenous methylprednisolone and prednisolone shows that 20% of patients have an unusual kinetic without an identifiable cause. Other findings included different absorption of GCs and reduced clearance with older age [78].

It is not clear whether or not the dose and the duration of GCs therapy increase the risk of developing adrenal insufficiency: some authors found no relationship between either the dose or duration of therapy and adrenal insufficiency [13], while others found that both cumulative doses and treatment durations are associated with an increased risk of adrenal insufficiency [79].

Even if severe complications, such as adrenal crisis, are rare in central adrenal insufficiency, this possibility has to be considered in patients with tertiary adrenal insufficiency, in particular if there is an acute stress (e.g., surgical or infectious stress).

In their paper, Smans et al. retrospectively evaluated the incidence of adrenal crisis in a group of patients affected by adrenal insufficiency in a period of 30 years and try to identify associated risk factors. Tertiary adrenal insufficiency is present, as mentioned before, in the 6% of observed patients (28 patients) and it has been induced by the chronic GC use for inflammatory diseases, chronic inhalation GCs, or chronic topical corticosteroid use. Interestingly, the incidence rate of adrenal crisis is 15.1/100 patients-year (PY) in tertiary adrenal insufficiency, while it is lower (and comparable to previous reported studies) for primary (5.2/100 PY) and secondary (3.6/100 PY) adrenal insufficiency. Considering all patients, the most important precipitating factor for adrenal crisis are infections (mostly gastroenteritis and bronchopulmonary infection) but, in about the 30% of patients with tertiary adrenal insufficiency, the crisis occurred after GC dose reduction [35].

The clinical paradox often present in patients treated with synthetic GCs is that both signs and symptoms of hypercortisolism and clinical and biochemical features of suppression of HPA axis and adrenal insufficiency, in particular after abrupt cessation or too rapid withdrawal of GCs, can be present. Thus, the most important challenge is to identify patients with suspected HPA axis suppression. As mentioned before, any type of GCs formulation is not risk-free. Certainly the potency, the dose, and the duration of GC use are important, but only approximate, predictors of the presence of HPA suppression. Long-acting preparations have a longer tissue life which induces a chronic state of hypercortisolism, making HPA axis suppression more likely [12]: indeed, hydrocortisone and cortisone acetate are the least suppressive agents, while prednisone, prednisolone, methylprednisolone, and triamcinolone are moderately suppressive. Dexamethasone is the most potent ACTH suppressor (as demonstrated also by its use in the diagnosis of Cushing’s syndrome). Furthermore, systemic therapy is more likely to suppress the HPA axis, even if other routes of administration (inhalation, topical, intra-ocular) cause HPA axis suppression depending on the bioavailability of the drug [80].

Accordingly to the literature [81], in patients who have received a GC dose comparable with more than 20 mg of prednisone/day for more than three weeks, HPA suppression is “likely”. However, this is true also for patients who have received an evening/bedtime dose of ≥5 mg of prednisone for more than a few weeks. In fact, evening doses of GCs tend to suppress the normal early morning surge of ACTH secretion, resulting in greater adrenal suppression. Furthermore, any patient who has a Cushingoid appearance is at risk to develop tertiary hypoadrenalism. In these patients, it may be not necessary to perform tests to evaluate HPA function, but they should be treated as if affected by central hypoadrenalism. Patients with intermediate or uncertain risk of HPA suppression include those who are taking 10 to 20 mg of prednisone/day for more than three weeks or any patient who has taken any dose < 10 mg of prednisone/day for more than a few weeks (but not as a single bedtime dose). In case of GCs withdrawal, a gradual reduction in doses is appropriate for these patients; an HPA functional test is not necessary, unless abrupt discontinuation should be considered or patients are at risk of acute stress (for example, surgery).

Duration and cumulative doses of GC treatment are also useful in defining HPA suppression [82], which is unlikely if the treatment has been performed for less than three weeks (any dose) or with an alternate-day prednisone at a dose of less than 10 mg. In fact, there is a clear evidence that patients are at lower risk for adrenal insufficiency if they take GCs on alternate days from the outset or if they can convert to alternate-day therapy before the development of HPA axis suppression [83].

However, individual variation in HPA axis function after GC withdrawal can be observed and it may be caused by individual variation in sensitivity towards GCs. It has been demonstrated that the risk of suppressed adrenal function after a seven-day prednisone treatment is increased in subjects who have a low cortisol level after a dexamethasone suppression test [84]. Individual differences in GC sensitivity are important in evaluating a patient’s risk of developing GC-induced adrenal insufficiency. The underlying mechanisms of action can be different, ranging from pharmacokinetic and pharmacodynamic properties of the used drugs to genetic predisposition [85]. Several single nucleotide polymorphisms (SNPs), occurring with high frequency and involving GABA receptors, opioid receptors, α2-adrenergic receptors, serotonin transporter, catechol *O*-methyltransferase, MR, and GR have been associated with the changes in HPA axis reactivity [86]. The majority of GR polymorphisms is connected with a loss of function and often with GC resistance [87], but there are also gain-of function GR polymorphisms. For example, the isoform named hGR DL-2 has a decrease in transactivation potential of more than 90%. On the other hand, the SNP A829G, was found to increase the transactivation potential of the GR [88]. The presence of either SNP A214G or T962C results in a decreased response after stimulation with both hydrocortisone and dexamethasone [89].

## 4. Conclusions

Despite steroid efficacy in different conditions (rheumatic, pulmonary, gastroenterological, and cutaneous diseases), the side effects induced by synthetic GCs generally require tapering of the drug as soon as the underlying disease is under control. However, tapering must be done carefully to avoid not only recurrent activity of the underlying disease, but also the possible cortisol deficiency resulting from HPA axis suppression. The effects of synthetic GCs are mediated by their binding to GRs and MRs. HPA axis function is based on the well-known negative feedback mechanism, which seems to involve in particular GRs mainly localized in the pituitary. Several factors regulate the degree of inhibition of HPA axis during synthetic GCs therapy, such as the type of used compound and the method of administration, the cumulative dose, and the duration of the treatment, as well as the possible concomitant use of drugs which can increase the bioavailability of GCs. In addition of these elements, there is a considerable variation in individual physiology which might explain why some patients exhibit changes in HPA-axis function while others do not. The discovery that there are multiple GR isoforms generated by alternative splicing [90] leads to understanding the molecular basis for this different sensitivity to GC [91].

Therefore, the challenge of future research is to evaluate the impact of these different genetic profiles on the clinical practice.

## Figures and Tables

**Table 1:** Most used synthetic glucocorticoids and their characteristics [12]. Anti-inflammatory activity and mineralocorticoid activity of each compound are related to those of hydrocortisone, which is 1. The equivalent dose is expressed in mg.

Synthetic Glucocorticoids	Equivalent Dose (mg)	Anti-Inflammatory Activity (Related to Hydrocortisone)	Mineralocorticoid Activity (Related to Hydrocortisone)	Biological Half-Life (hours)
Hydrocortisone	20	1	1	8–12
Cortisone Acetate	25	0.8	0.8	8–12
Deflazacort	5	4	1	<12
Prednisone	5	4	0.3	12–36
Prednisolone	5	4	0.3	12–36
Triamcinolone	4	5	0	12–36
Methylprednisolone	4	5	0.5	12–36
Paramethasone	2	10	0	/
Dexamethasone	0.75	30	0	36–72
Betamethasone	0.6	25	0	36–72
Fludrocortisone	Not for anti-inflammatory use	10	250	18–36

**Table 2:** Inhaled synthetic glucocorticoids [12,39].

Inhaled synthetic Glucocorticoids	Receptor Binding Affinity (Relative to Dexamethasone = 1)	Oral Bioavailability (%)	Systemic Clearance (L/h)	Half-Life (h)
Beclomethasone dipropionate	0.4	20	150	Unknown
Beclomethasone 17-monopropionate	13.5	40	120	2.7
Budesonide	9.4	11	84	2.0
Ciclesonide	0.12	<1	152	0.5
Flunisolide	1.8	20	58	1.6
Fluticasone propionate	18	≤1	66	14.4
Mometasone furoate	23	<1	53	Unknown
Triamcinolone acetonide	3.6	23	45	3.6

**Table 3:** Topical synthetic glucocorticoids (potency according to European Corticosteroids Classification) [48].

Potency	Topical Synthetic Glucocorticoids
Low	Hydrocortisone acetate 1% Alclometasone dipropionate 0.05% Methylprednisolone acetate 0.25%
Medium	Clobetasone butyrate 0.05% Hydrocortisone butyrate 0.1% Fluocortolone pivalate 0.5%
High	Beclomethasone dipropionate 0.025% Betamethasone dipropionate 0.05% Betamethasone benzoate 0.025% Betamethasone valerate 0.1% Difluocortolone valerate 0.1% Fluocinolone acetonide 0.025% Fluticasone propionate 0.05% Fluocinonide 0.05%
Very High	Clobetasol propionate 0.05% Diflucortolone valerate 0.3% Halcinonide 0.01%

## Abbreviations

11β-HSD2	11-β-hydroxysteroid dehydrogenase type 2
ACTH	Adrenocorticotropic hormone
AVP	Arginine vasopressin
CBG	Corticosteroid binding globulin
CRH	Corticotropin-release hormone
cAMP	Cyclic adenosine monophosphate
GABA	Gamma-aminobutyric acid
GC	Glucocorticoid
GR	Glucocorticoid receptor
HPA	Hypothalamus–pituitary–adrenal
KO	Knockout
MC2R	Melanocyte type-2 receptor
MC2R	Mineralocorticoid
MR	Mineralocorticoid receptor
VO	Nitric oxide
PVN	Paraventricular nucleus
PY	Patients-year
POMC	Proopiomelanocortin
RTV	Ritonavir
SEGRAs	Selective GC receptor agonists
SNPs	Single nucleotide polymorphisms
StAR	Steroidogenic acute regulatory
SCN	Suprachiasmatic nucleus
